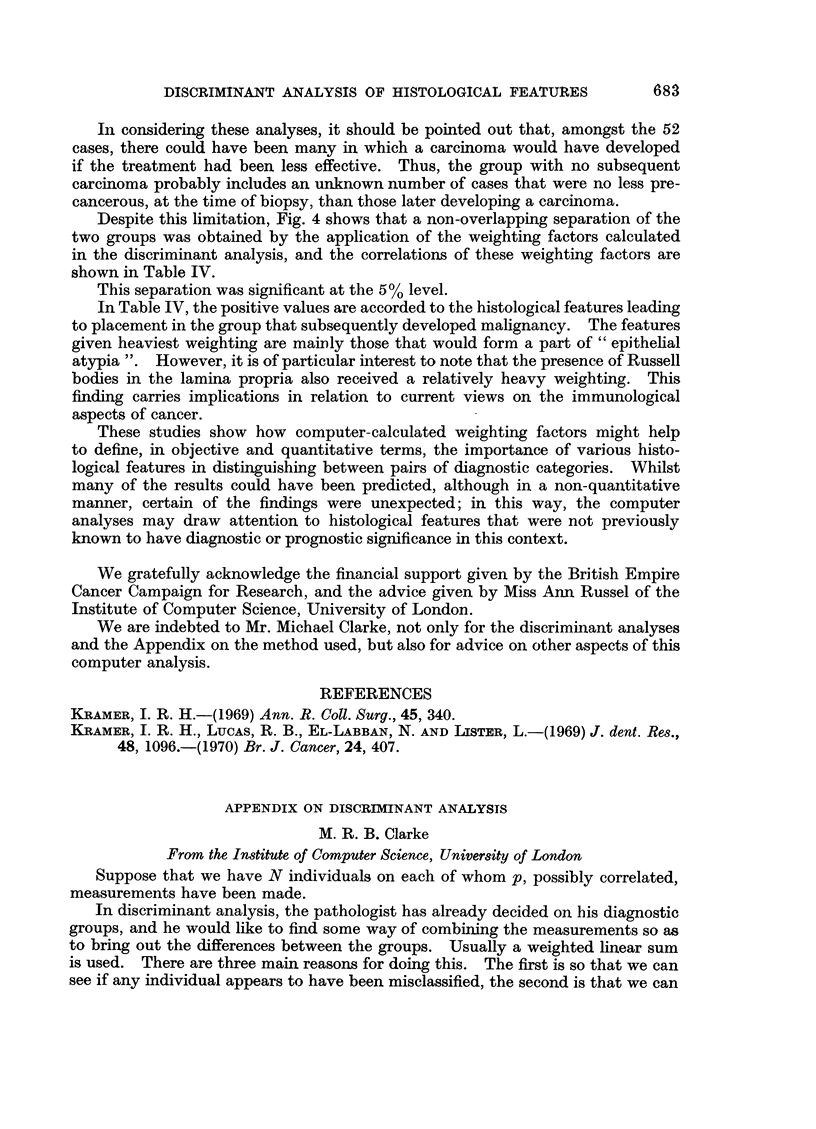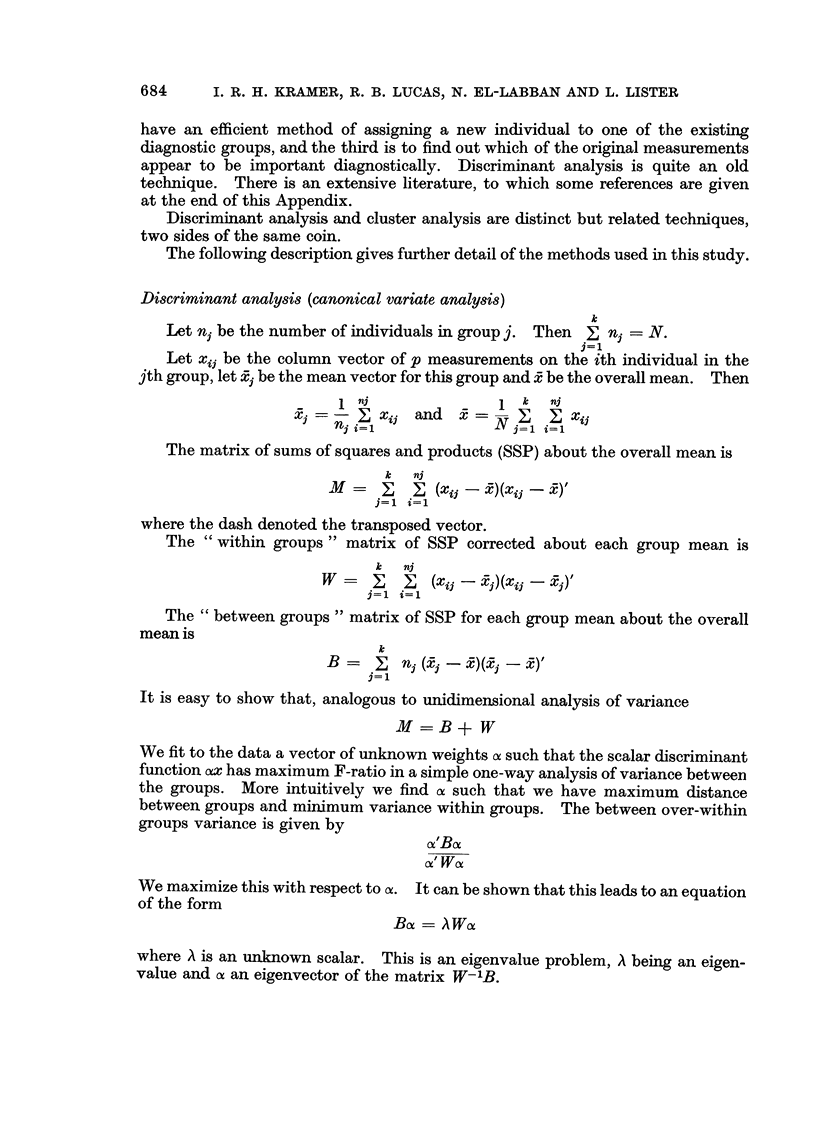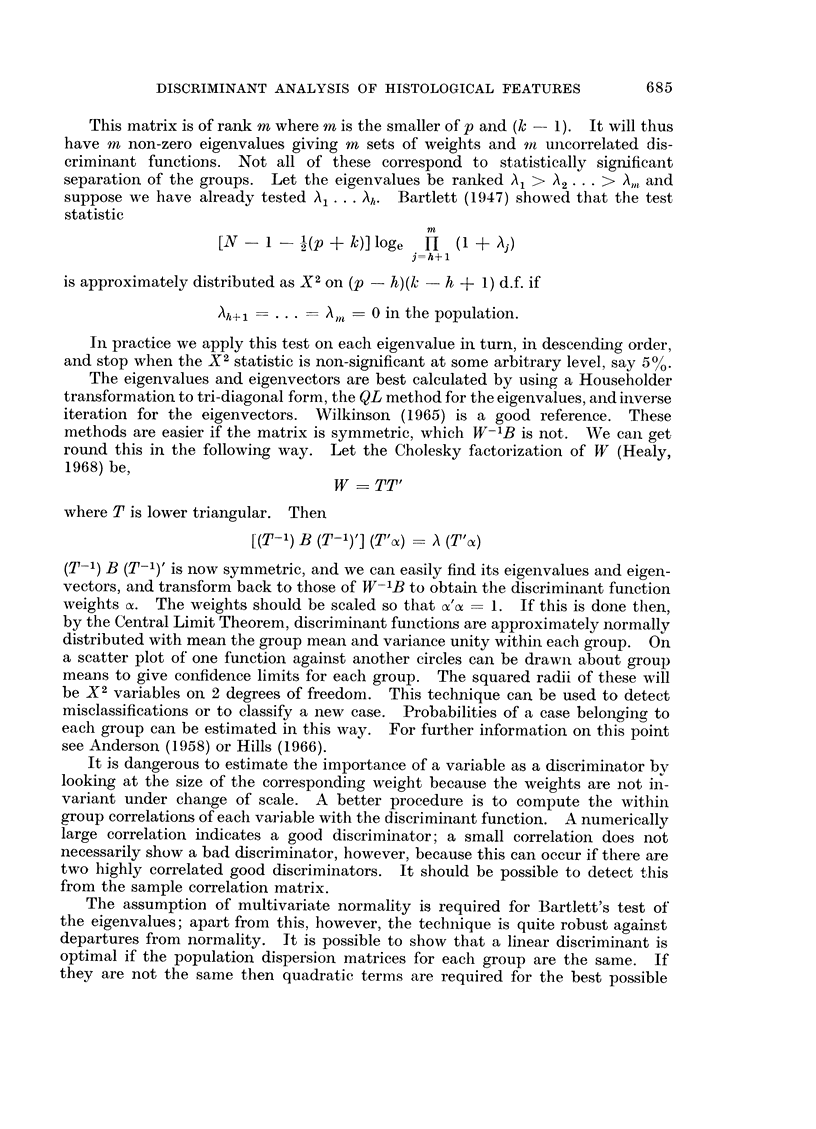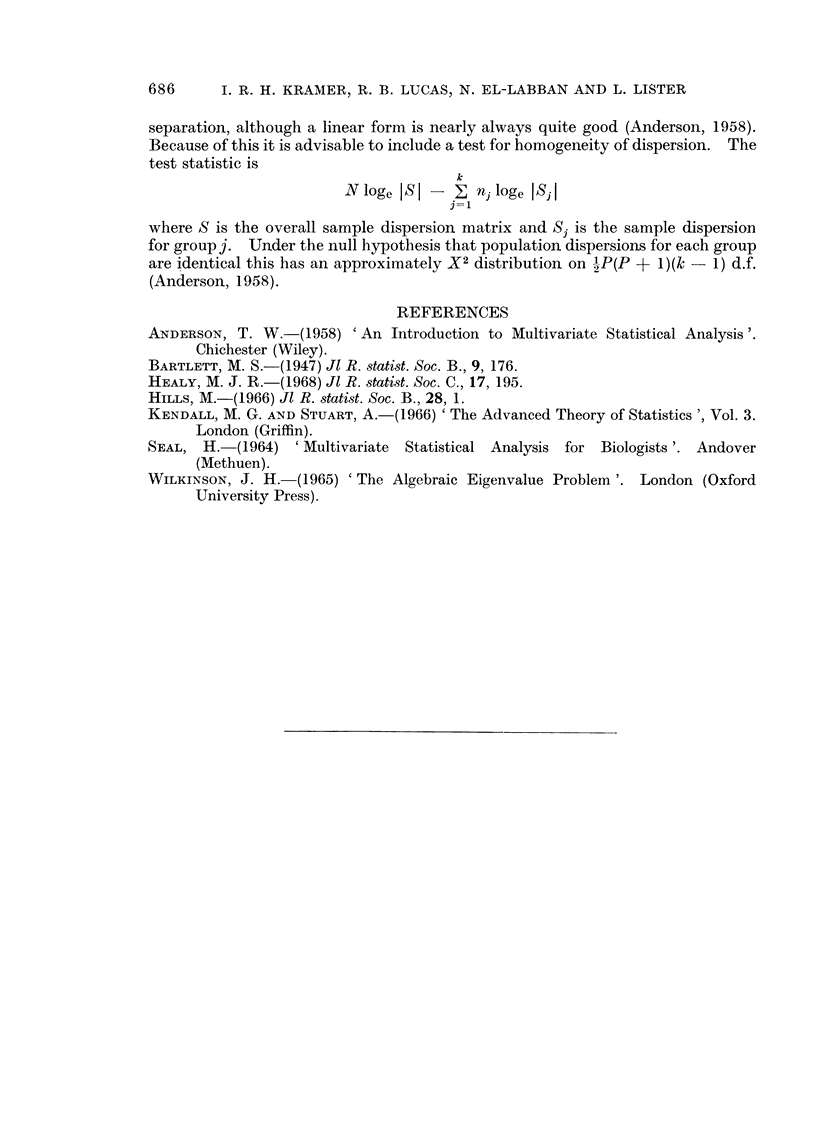# The use of discriminant analysis for examining the histological features of oral keratoses and lichen planus. Appendix on discriminant analysis.

**DOI:** 10.1038/bjc.1970.81

**Published:** 1970-12

**Authors:** M. R. Clarke


### APPENDIX ON DISCRIMINANT ANALYSIS

M. R. B. Clarke

*From the Institute of Computer Science, University of London*

Suppose that we have $N$ individuals on each of whom $p$, possibly correlated, measurements have been made.

In discriminant analysis, the pathologist has already decided on his diagnostic groups, and he would like to find some way of combining the measurements so as to bring out the differences between the groups. Usually a weighted linear sum is used. There are three main reasons for doing this. The first is so that we can see if any individual appears to have been misclassified, the second is that we can have an efficient method of assigning a new individual to one of the existing diagnostic groups, and the third is to find out which of the original measurements appear to be important diagnostically. Discriminant analysis is quite an old technique. There is an extensive literature, to which some references are given at the end of this Appendix.

Discriminant analysis and cluster analysis are distinct but related techniques, two sides of the same coin.

The following description gives further detail of the methods used in this study.

*Discriminant analysis (canonical variate analysis)*

Let $n_j$ be the number of individuals in group $j$. Then $\sum_{j=1}^{k} n_j = N$.

Let $x_{ij}$ be the column vector of $p$ measurements on the $i$th individual in the $j$th group, let $\bar{x}_j$ be the mean vector for this group and $\bar{x}$ be the overall mean. Then

$$\bar{x}_j = \frac{1}{n_j} \sum_{i=1}^{nj} x_{ij} \quad \text{and} \quad \bar{x} = \frac{1}{N} \sum_{j=1}^{k} \sum_{i=1}^{nj} x_{ij}$$

The matrix of sums of squares and products (SSP) about the overall mean is

$$M = \sum_{j=1}^{k} \sum_{i=1}^{nj} (x_{ij} - \bar{x})(x_{ij} - \bar{x})'$$

where the dash denoted the transposed vector.

The " within groups " matrix of SSP corrected about each group mean is

$$W = \sum_{j=1}^{k} \sum_{i=1}^{nj} (x_{ij} - \bar{x}_j)(x_{ij} - \bar{x}_j)'$$

The " between groups " matrix of SSP for each group mean about the overall mean is

$$B = \sum_{j=1}^{k} n_j (\bar{x}_j - \bar{x})(\bar{x}_j - \bar{x})'$$

It is easy to show that, analogous to unidimensional analysis of variance

$$M = B + W$$

We fit to the data a vector of unknown weights $\alpha$ such that the scalar discriminant function $\alpha x$ has maximum F-ratio in a simple one-way analysis of variance between the groups. More intuitively we find $\alpha$ such that we have maximum distance between groups and minimum variance within groups. The between over-within groups variance is given by

$$\frac{\alpha' B \alpha}{\alpha' W \alpha}$$

We maximize this with respect to $\alpha$. It can be shown that this leads to an equation of the form

$$B\alpha = \lambda W \alpha$$

where $\lambda$ is an unknown scalar. This is an eigenvalue problem, $\lambda$ being an eigenvalue and $\alpha$ an eigenvector of the matrix $W^{-1}B$.

This matrix is of rank $m$ where $m$ is the smaller of $p$ and $(k - 1)$. It will thus have $m$ non-zero eigenvalues giving $m$ sets of weights and $m$ uncorrelated discriminant functions. Not all of these correspond to statistically significant separation of the groups. Let the eigenvalues be ranked $\lambda_1 > \lambda_2 \ldots > \lambda_m$ and suppose we have already tested $\lambda_1 \ldots \lambda_h$. Bartlett (1947) showed that the test statistic

$$[N - 1 - \tfrac{1}{2}(p + k)] \log_e \prod_{j=h+1}^{m} (1 + \lambda_j)$$

is approximately distributed as $X^2$ on $(p - h)(k - h + 1)$ d.f. if

$$\lambda_{h+1} = \ldots = \lambda_m = 0 \text{ in the population.}$$

In practice we apply this test on each eigenvalue in turn, in descending order, and stop when the $X^2$ statistic is non-significant at some arbitrary level, say 5%.

The eigenvalues and eigenvectors are best calculated by using a Householder transformation to tri-diagonal form, the $QL$ method for the eigenvalues, and inverse iteration for the eigenvectors. Wilkinson (1965) is a good reference. These methods are easier if the matrix is symmetric, which $W^{-1}B$ is not. We can get round this in the following way. Let the Cholesky factorization of $W$ (Healy, 1968) be,

$$W = TT'$$

where $T$ is lower triangular. Then

$$[(T^{-1}) B (T^{-1})'] (T'\alpha) = \lambda (T'\alpha)$$

$(T^{-1}) B (T^{-1})'$ is now symmetric, and we can easily find its eigenvalues and eigenvectors, and transform back to those of $W^{-1}B$ to obtain the discriminant function weights $\alpha$. The weights should be scaled so that $\alpha'\alpha = 1$. If this is done then, by the Central Limit Theorem, discriminant functions are approximately normally distributed with mean the group mean and variance unity within each group. On a scatter plot of one function against another circles can be drawn about group means to give confidence limits for each group. The squared radii of these will be $X^2$ variables on 2 degrees of freedom. This technique can be used to detect misclassifications or to classify a new case. Probabilities of a case belonging to each group can be estimated in this way. For further information on this point see Anderson (1958) or Hills (1966).

It is dangerous to estimate the importance of a variable as a discriminator by looking at the size of the corresponding weight because the weights are not invariant under change of scale. A better procedure is to compute the within group correlations of each variable with the discriminant function. A numerically large correlation indicates a good discriminator; a small correlation does not necessarily show a bad discriminator, however, because this can occur if there are two highly correlated good discriminators. It should be possible to detect this from the sample correlation matrix.

The assumption of multivariate normality is required for Bartlett's test of the eigenvalues; apart from this, however, the technique is quite robust against departures from normality. It is possible to show that a linear discriminant is optimal if the population dispersion matrices for each group are the same. If they are not the same then quadratic terms are required for the best possible separation, although a linear form is nearly always quite good (Anderson, 1958). Because of this it is advisable to include a test for homogeneity of dispersion. The test statistic is

$$N \log_e |S| - \sum_{j=1}^{k} n_j \log_e |S_j|$$

where $S$ is the overall sample dispersion matrix and $S_j$ is the sample dispersion for group $j$. Under the null hypothesis that population dispersions for each group are identical this has an approximately $X^2$ distribution on $\frac{1}{2}P(P + 1)(k - 1)$ d.f. (Anderson, 1958).

### REFERENCES

ANDERSON, T. W.—(1958) 'An Introduction to Multivariate Statistical Analysis'. Chichester (Wiley).
BARTLETT, M. S.—(1947) *Jl R. statist. Soc.* B., **9**, 176.
HEALY, M. J. R.—(1968) *Jl R. statist. Soc.* C., **17**, 195.
HILLS, M.—(1966) *Jl R. statist. Soc.* B., **28**, 1.
KENDALL, M. G. AND STUART, A.—(1966) 'The Advanced Theory of Statistics', Vol. 3. London (Griffin).
SEAL, H.—(1964) 'Multivariate Statistical Analysis for Biologists'. Andover (Methuen).
WILKINSON, J. H.—(1965) 'The Algebraic Eigenvalue Problem'. London (Oxford University Press).